# Pharmacological Inhibition of Inositol-Requiring Enzyme 1α RNase Activity Protects Pancreatic Beta Cell and Improves Diabetic Condition in Insulin Mutation-Induced Diabetes

**DOI:** 10.3389/fendo.2021.749879

**Published:** 2021-10-05

**Authors:** Oana Herlea-Pana, Venkateswararao Eeda, Ram Babu Undi, Hui-Ying Lim, Weidong Wang

**Affiliations:** ^1^ Department of Medicine, Division of Endocrinology, Harold Hamm Diabetes Center, Oklahoma City, OK, United States; ^2^ Department of Physiology, Harold Hamm Diabetes Center, The University of Oklahoma Health Science Center, Oklahoma City, OK, United States

**Keywords:** Beta cell failure, beta cell protection, ER stress, Ire1alpha, Ire1alpha inhibition, unfolded protein response, monogenic diabetes, proinsulin misfolding

## Abstract

β-cell ER stress plays an important role in β-cell dysfunction and death during the pathogenesis of diabetes. Proinsulin misfolding is regarded as one of the primary initiating factors of ER stress and unfolded protein response (UPR) activation in β-cells. Here, we found that the ER stress sensor inositol-requiring enzyme 1α (IRE1α) was activated in the Akita mice, a mouse model of mutant insulin gene-induced diabetes of youth (MIDY), a monogenic diabetes. Normalization of IRE1α RNase hyperactivity by pharmacological inhibitors significantly ameliorated the hyperglycemic conditions and increased serum insulin levels in Akita mice. These benefits were accompanied by a concomitant protection of functional β-cell mass, as shown by the suppression of β-cell apoptosis, increase in mature insulin production and reduction of proinsulin level. At the molecular level, we observed that the expression of genes associated with β-cell identity and function was significantly up-regulated and ER stress and its associated inflammation and oxidative stress were suppressed in islets from Akita mice treated with IRE1α RNase inhibitors. This study provides the evidence of the *in vivo* efficacy of IRE1α RNase inhibitors in Akita mice, pointing to the possibility of targeting IRE1α RNase as a therapeutic direction for the treatment of diabetes.

## Highlights

- Proinsulin misfolding in the endoplasmic reticulum (ER) plays an important role in beta cell dysfunction and death and the pathogenesis of mutant *INS*-gene-induced diabetes of youth (MIDY).
- ER stress activates unfolded protein response (UPR) including IRE1α pathway.
- It is unknown whether inhibition of IRE1α RNase activity can protect beta cells and improve diabetic conditions in MIDY animals.
- Pharmacological inhibition of IRE1α RNase lowers blood glucose levels and increases serum insulin levels in diabetic animals.
- IRE1α inhibition protects beta cell function and survival.
- IRE1α inhibition suppresses ER stress-associated inflammation and oxidative stress.
- Targeting IRE1α RNase may provide a potential effective therapeutic for the treatment of diabetes.

## Introduction

Endoplasmic reticulum (ER) stress is a condition in which unfolded or misfolded proteins accumulate in the ER. Upon ER stress, the unfolded protein response (UPR) is activated to initially serve as an adaptive means to resolve ER stress, but eventually becomes maladaptive when activated chronically, leading to cellular dysfunction and death (1). The UPR is transduced by three core pathways – inositol requiring enzyme 1-α (IRE1-α), activating transcription factor 6 (ATF6), and PKR-like ER kinase (PERK) (1). IRE-1α, the most evolutionarily conserved among the UPR sensors, is an ER transmembrane protein with dual serine/threonine kinase and RNase domains. Binding of misfolded proteins to IRE1α luminal domain leads to its aggregation, thereby eliciting the sequential activation of its kinase and RNase domains (2–5). IRE1α hyperactivation has been observed to contribute to pathological manifestation and progression (6–9) in multiple diseases, and overexpression of IRE1α alone is sufficient to cause cell death (10, 11). As ER stress in multiple cell types including β-cells contributes to diabetes pathogenesis, targeting IRE1α or ER stress has been proposed as a potential therapeutic option for diabetes (5, 12). Several kinase inhibitors were recently reported to protect β cells by inhibiting IRE1α kinase activity (11, 13, 14); however, subsequent studies revealed that these molecules likely act on other cellular targets to accomplish their biological activities (15–22). Therefore, it remains unclear whether IRE1α inhibition is protective in β cells under ER stress.

On the other hand, IRE1α plays an important role in maintaining ER homeostasis under both physiological settings and the early adaptive phase of ER stress (6, 23–28). The IRE1α/XBP1 axis is crucial for ER expansion in secretory cells such as plasma cells (29) and prevents ER membrane permeabilization and ER stress-induced cell death under pathological conditions (24). In β-cells, IRE1α also critically regulates postprandial insulin biosynthesis, proinsulin folding, and insulin secretion (23, 28, 30, 31), As a corollary, IRE1α knockout β-cells exhibited functional impairments (31, 32). Together, these findings support an important physiological role of IRE1α and raise the question as to whether inhibiting IRE1α represents a viable approach in countering ER stress-related pathological diseases.

β-cell dysfunction and death is an important aspect in the pathogenesis of all forms of diabetes (33–37). In β-cells, proinsulin is misfolding-prone even under normal physiologic condition (35, 38–40) and proinsulin misfolding is regarded as one of the primary initiating factors of ER stress in β-cells (41–43). The autosomal-dominant diabetes known as Mutant *INS*-gene-induced Diabetes of Youth (MIDY) (33, 37) manifests proinsulin misfolding and progressive β-cell dysfunction and death (33–37), and therefore is an ideal model to study the effect of IRE1α in β-cell function and survival and in diabetes control. In this study, we report, for the first time, the effect of IRE1α RNase inhibitors on the diabetic conditions and β-cells in *Akita* mouse (44–46), an animal model of MIDY. We showed that IRE1α RNase is activated in Akita islets and that treating *Akita* mice with IRE1α RNase inhibitors significantly lowers blood glucose levels and increases serum insulin levels. These effects are accompanied by functional β-cell preservation. Finally, ER stress and associated oxidative stress and inflammation in β-cells are suppressed. Collectively, these studies serve as a foundation for targeting IRE1α as a therapeutic means in the treatment of diabetes.

## Material and Methods

### Animal Studies

C57BL/6J wild-type (WT) mice and Akita mice were obtained from Jackson laboratory (Bar Harbor, ME). The genotyping of Akita mice was confirmed using tetra-primer ARMS-PCR approach (46). Mice were housed on a 12 h light (6:00 a.m. to 6:00 p.m.)−12 h dark (6:00 p.m. to 6:00 a.m.) cycle at an ambient temperature of 22°C and fed normal chow diet and water ad libitum. All procedures involving animals were performed in accordance with the protocol approved by the Institutional Animal Care and Use Committee of the University of Oklahoma Health Science Center. All experiments were performed with age-matched female mice.

Akita mice at 5-6 weeks of age were randomly grouped for the injection i.p. with either vehicle (*n* = 9 mice), STF (10 mg/kg body weight; 2 mg/ml in 10% DMSO in saline buffer; *n* = 9 mice) or 4μ8C (10 mg/kg of body weight) once daily. These doses were chosen based on previously reported efficacy shown on mice *via* IP injection (47, 48). Compounds were dosed approximately 3−4 h before the initiation of the dark cycle (2−3 p.m.). Blood glucose levels were measured using the OneTouch Ultra2 glucometer after fasting for 6 h. Body weights were measured weekly. At the end of treatment, mice were fasted for 4 h and euthanized, and pancreata were removed and weighted. A tail end portion of the pancreata was saved for insulin and proinsulin content measure while the remaining pancreata were formalin fixed and paraffin-embedded.

### Glucose Tolerance Test and Insulin Tolerance Test

Intraperitoneal glucose tolerance test (ipGTT) and intraperitoneal insulin tolerance test (ipITT) were performed after 16-h and 4-hour fasting, respectively. Blood glucose levels were measured at 0, 15, 30, 60, and 120 minutes after intraperitoneal administration of glucose (1.5 g/kg body weight) for ipGTT or insulin (0.75 IU/kg body weight) for ipITT.

### Islet Isolation Procedure

Islets were isolated using the standard collagenase digestion method. Briefly, the common bile duct was cannulated and distended with Collagenase P (0.5 mg/ml, Sigma-Aldrich, USA) in 1x Hank’s balanced salt solution. Pancreata were removed and incubated in water bath at 37C for 25 m. Islets were separated using Histopaque-1077 (Sigma-Aldrich, USA) and cultured overnight at 37°C in RPMI1640 media containing 10% FBS.

### Islet Western Blotting for Proinsulin Misfolding

Islets isolated from 6-week Akita or WT B/6J mice were treated with STF 20 μM or DMSO vehicle (0.1%) for 3 h. Proteins were extracted with RIPA buffer (10 mM Tris pH 7.4, 150 mM NaCl, 0.1% SDS, 1% NP40, 2 mM EDTA) plus protease inhibitor/phosphatase inhibitor cocktail (Sigma-Aldrich) and centrifuged at 4°C for 10 min at 10,000 g. Total protein concentration in the cell lysate was determined by BCA. Samples of ~ 20 μg protein prepared in Laemmli sample buffer without (non-reducing) or with (reducing) 5% b-mercaptoethanol were resolved on 4-12% Bis-Tris NuPAGE gels (Invitrogen) at 100 V for 60 min. The nonreducing gels were incubated in 25 mM dithiothreitol (DTT) solution for 10 min at room temperature before being transferred to PVDF membranes for 25 min at 4°C at 40 V. Membranes were probed with anti-proinsulin antibody (CCI-17, NOVUS) and HRP-conjugated secondary antibodies (1:3000; Santa Cruz Biotechnology, CA, USA).

### RNA Isolation and RT-PCR

Total RNA was extracted using TRIzol reagent (Invitrogen, Carlsbad, CA) according to the manufacturer’s protocol. 2 μg of total RNA was reverse transcribed using Superscript kit (Invitrogen). Real-time PCR was performed with a CFX96 Real-Time PCR detection system (Bio-Rad, CA) using SYBR Select Master Mix (Applied Biosystems, CA). Relative mRNA levels were normalized against Cyclophilin A. Primers used are shown in **Table S1**. Regular RT-PCR was performed and the products was resolved by agarose gel electrophoresis. The unspliced and spliced XBP1 mRNA levels were quantified using ImageJ software (National Institutes of Health, Bethesda, MD).

### Glucose-Stimulated Insulin or Proinsulin Secretion

20 primary islets isolated from Akita mice treated with STF or vehicle were seeded in 96-well plates overnight and then incubated in fresh KRBH buffer (115 mM NaCl, 5 mM KCl, 24 mM NaHCO_3_, 2.5 mM CaCl_2_, 1 mM MgCl_2_, 10 mM HEPES, 2% w/v BSA, pH 7.4) containing 2.5 mM glucose for 1 h. Islets were incubated for an additional hour in KRBH buffer containing 2.5 or 16.7 mM glucose. Secreted insulin and proinsulin levels were measured with insulin ELISA kits (ALPCO, Salem, NH) and Rat/Mouse Proinsulin kit (Mercodia), respectively and normalized to total protein of cell lysates.

### Insulin and Proinsulin Content Measurements

Pancreatic tissues or islets were incubated and homogenized in 1.5% HCl in 70% EtOH overnight at -20C, and the solution neutralized with equal volume of 1M Tris pH 7.5. Insulin and proinsulin were measured by ELISA kits as outlined above and normalized to weights of pancreas for pancreatic tissues or to protein levels for islets.

### Immunofluorescent Staining and Islet Mass Measurement

Pancreata were fixed in formalin and paraffin-embedded. 6-8 slide sections on average from each mouse for all mice were sectioned with the separation at 150 μm increments. Images covering the entire tissue sample were captured in each section. The entire pancreas tissue, glucagon^+^, and insulin^+^ areas in each image were measured using ImageJ software. Relative β-cell area = sum of all islet β-cell areas/sum of the total pancreatic area, and normalized against the β-cell area of WT B/6J mice (set as 1). All images were taken with an Olympus FV1000 confocal microscope and quantified with Image-J histogram software.

Antibodies used for staining: GP anti-insulin antibody (A0564, 1:500; Dako), mouse anti-glucagon antibody (G2654, 1:500; Sigma), mouse anti-caspase 3 (cat# 9446, 1:500, CST), rabbit anti-Ki67 (Ab15580, 1:250, Abcam), mouse anti-proinsulin (GS-9A8, 1:100, DSHB), Anti-4-Hydroxynonenal [HNEJ-2] (ab48506, Abcam), DAPI (0.5 μg/mL), and Alexa Fluor 488-, 555-, and 647-conjugated secondary antibodies (Jackson ImmunoResearch).

### TUNEL Staining

TUNEL staining was performed together with antibodies as above in pancreatic sections with In Situ Cell Death Detection Kit-Fluorescein (Roche) according to the manufacturer’s instructions.

### Transmission Electron Microscopy (TEM)

Isolated mouse islets were fixed with 0.1 M sodium phosphate buffer (pH 7.2) containing 2% glutaraldehyde and 2% paraformaldehyde for 1 h, then exposed to 2% osmium tetroxide, stained with 2% uranyl acetate, dehydrated with ethanol, and embedded in Epon (TAAB). Ultra-thin sections were stained with uranyl acetate and lead citrate, and images were recorded with a Hitachi H-7600 transmission electron microscope (Hitachi).

### Statistical Analysis

Data were analyzed using the unpaired two-tailed Student’s t-test or one-way ANOVA for multiple comparisons. All values are reported as mean ± SEM and p<0.05 was considered statistically significant.

## Results

### Up-Regulation of IRE1α RNase Activity in Pancreatic Islets in Akita Mice

**Figure 1:**
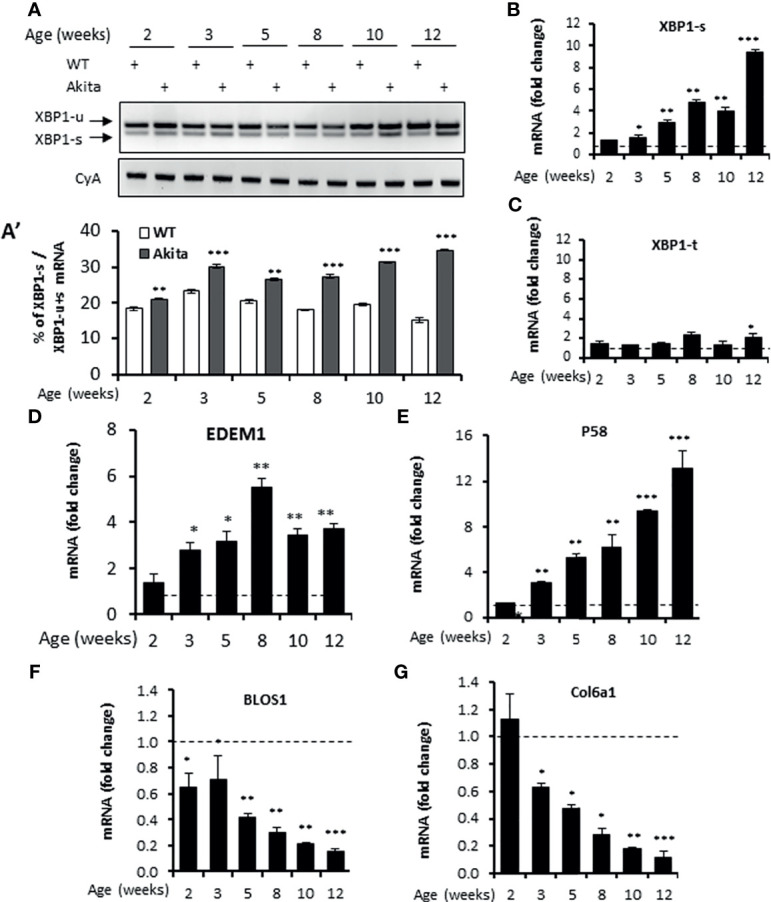
IRE1α RNase activity up-regulation in islets of Akita mice. **(A, A’)**. XBP1 mRNA levels were analyzed in islets isolated from Akita mice or age-matched C57B/6 mice at the indicated ages by RT-PCR, and the products were resolved by agarose gel electrophoresis. The full length (unspliced, XBP1-u) and spliced (XBP1-s) forms of XBP1 mRNA were indicated **(A)** and quantified **(A’)**. Cyclophilin A mRNA was used as an internal control. The data shown are representative of 3 independent experiments. **(B–G)** mRNA levels for indicated genes were analyzed in islets isolated from Akita mice or age-matched C57B/6 mice by qRT-PCR. The results are expressed as the fold change over mRNA levels in respective age-matched controls (represented by the dashed line) and are representative of 3 independent experiments. *P < 0.05, **P < 0.01, and ***P < 0.001. Bars indicate SEM.

The C96Y missense mutation in the Ins2 gene in Akita mice causes mutant proinsulin protein misfolding that is responsible for ER stress (35). Previously, the ER stress response markers PERK and ATF6 have been reported to be up-regulated in *in vitro* β-cell lines carrying the Ins2^Akita/+^ mutation or in islets freshly isolated from Akita mice (44, 49, 50). Consistent with this, we observed that the mRNA levels of the PERK pathway genes *ATF4* and *CHOP* and the ATF6 target gene *Bip* were up-regulated in islets freshly isolated from Akita mice (herein terms Akita islets) over the age of 3 weeks (**Figures S1A–C**). However, how IRE-1α responds to this mutation in β-cells *in vivo* is unclear. Earlier studies using *in vitro* β-cell lines yielded controversial results, with one report showing an activation of the IRE-1α-XBP1 pathway in Ins2^Akita/+^ β-cell lines (51) and another report showing a down-regulation of IRE-1α activity in stable β-cell lines expressing Ins2^Akita/+^ mutation (52). To investigate the *in vivo* IRE-1α activity in Akita islets, we first examined the splicing of *Xbp1* mRNA, a direct target of IRE-1α RNase. We observed a gradual increase in *Xbp1* mRNA splicing in Akita islets from age of 2 weeks onwards compared to compared to the age-matched WT mice (**Figures 1A, A’**), as assessed by the electrophoretic separation of RT-PCR products. Similarly, quantitative RT-PCR also showed that spliced *Xbp1* (*Xbp1-s*) mRNA levels significantly increased in the Akita islets over a period of 12 weeks while total *Xbp1* (*Xbp1-t*) mRNA levels increased only slightly during the same period (**Figures 1B, C**). Second, we investigated the transcription of several XBP1 target genes *EDEM1* and *P58*, and observed a marked upregulation in their mRNA levels in Akita islets over WT islets (**Figures 1D, E**), as assessed by qRT-PCR. Third, as IRE-1α hyperactivation is associated with activation of IRE1-dependent decay of mRNA (RIDD) in which IRE1 cleaves mRNAs, we analyzed the mRNA levels of *Blos1* and *Col6a1*, two typical RIDD targets, by qRT-PCR. We observed that *Blos1* and *Col6a1* mRNA levels decreased progressively in the Akita islets from 3-week old onwards (**Figures 1F, G**). Together, our results demonstrate that IRE1α activity was already elevated at around 2 weeks of age, prior to the development of hyperglycemia in Akita mice, and continued to elevate until the Akita mice developed overt diabetes.

### Treatment of IRE1α RNase Inhibitor STF Ameliorates Diabetes in Akita Mice

**Figure 2:**
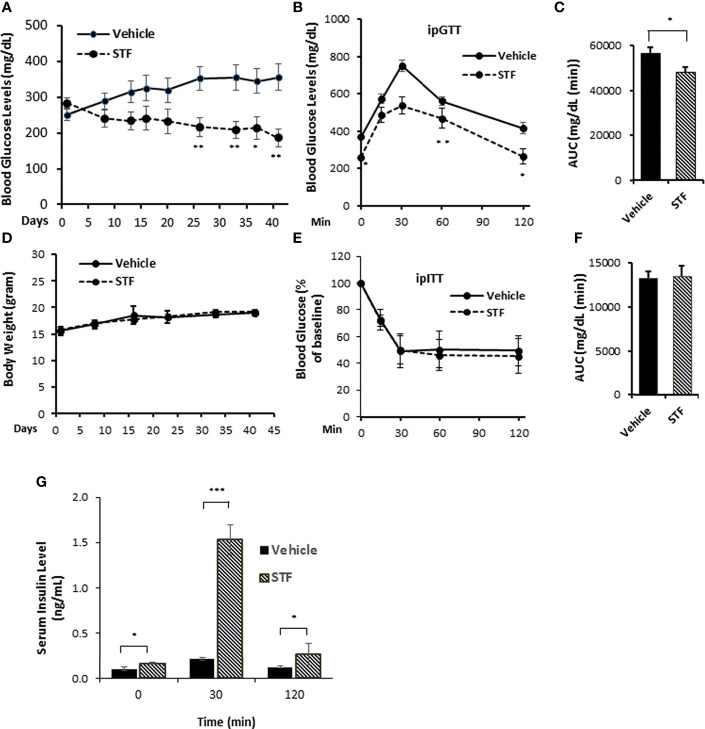
STF ameliorates diabetic conditions of Akita mice. **(A)** Fasting blood glucose levels were measured in Akita mice treated with vehicle (n = 8) or STF (n = 7) at indicated time points. **(B, C)** Glucose tolerance test. Blood glucose levels **(B)** measured at indicated time points after intraperitoneal injection of glucose (1.5g/kg body weight) following 6-h fasting and the AUC (area under the curve, **C**) at the end of 5-week treatment. **(D)** Body weight of mice. **(E, F)** Insulin tolerance test. Blood glucose levels **(E)** measured at indicated time points after intraperitoneal injection of insulin (0.75 IU/kg body weight) following 4-h fasting and the AUC a day before euthanization **(F)**. **(G)** In vivo glucose-stimulated insulin secretion. Serum insulin levels measured at indicated time points after intraperitoneal injection of glucose (1.5g/kg body weight) following 6-h fasting as in **(C)** *P < 0.05, **P < 0.01, and ***P < 0.001. Bars indicate SEM.

The results presented above in conjunction with previous observations that the overexpression of IRE-1α led to cell death in transfected cells (10, 53, 54) suggest that inhibiting IRE-1α may protect β-cells from Akita mutation-induced dysfunction and death and ameliorate the diabetic condition in Akita mice. We therefore investigated the effect of pharmacological inhibition of IRE-1α activity on β-cell and diabetic conditions in Akita mice. We treated Akita mice with a specific IRE-1α RNase inhibitor STF-083010 (STF, 10 mg/Kg of BW *via* IP injection, a dose previously shown to significantly inhibits IRE-1α RNase activity *in vivo*) (47, 55) for 6 weeks and detected a gradual and significant dampening of blood glucose levels over the treatment period (**Figure 2A**). In contrast, the vehicle-treated Akita mice continued to develop increasing hyperglycemia throughout the treatment period to reach up to 400mg/dL (**Figure 2A**). Furthermore, STF treatment significantly improved glucose tolerance and decreased AUC (area under the curve) in Akita mice compared to vehicle group (p<0.05; **Figures 2B, C**). On the other hand, the STF- and vehicle-treated Akita mice displayed comparable body weight (**Figure 2D**) and insulin sensitivity (**Figures 2E, F**), suggesting that STF lowers blood glucose levels not by altering insulin sensitivity. Finally, serum insulin levels in the STF-treated Akita mice were markedly increased compared to that of vehicle group; in particular, at 30 min after glucose injection (vehicle 0.2± 0.1 ng/ml vs. STF 1.5± 0.3 ng/ml; p<0.001) (**Figure 2G**). Together, these results indicate that STF treatment significantly alleviates the diabetic conditions in Akita mice.

### STF Treatment Attenuates IRE1-α Activity in Islets of Akita Mice

**Figure 3:**
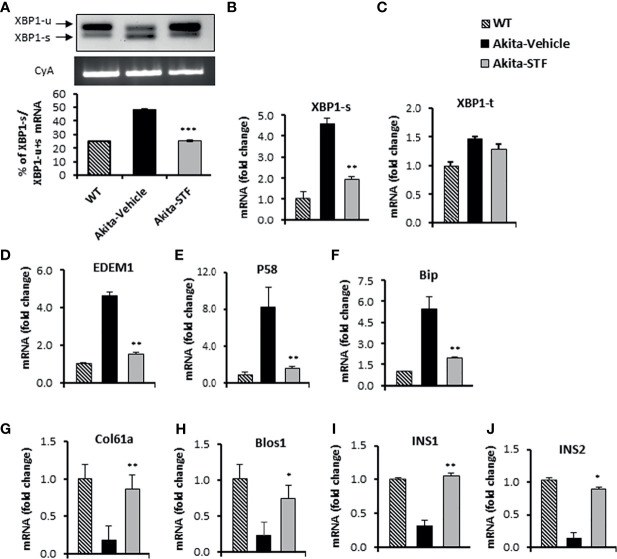
STF inhibits IRE1α RNase activity. **(A)** XBP1 mRNA levels were analyzed in islets isolated from Akita mice treated with STF or vehicle as in by RT-PCR and the products were resolved by agarose gel electrophoresis. The full length (unspliced, XBP1-u) and spliced (XBP1-s) forms of XBP1 mRNA were indicated and quantified by ImageJ program. Cyclophilin A mRNA was used as an internal control. **(B–J)** mRNA levels for indicated genes were analyzed in islets isolated from Akita mice treated with STF or vehicle by qRT-PCR. The results are expressed as fold change and are representative of 3 independent experiments. *P < 0.05, **P < 0.01, and ***P < 0.001 compared to Akita-vehicle group. Bars indicate SEM.

To investigate whether the STF amelioration of diabetic conditions in Akita mice is due to the inhibition of IRE1α activity in islets, we first examined the status of IRE1α-mediated *Xbp1* mRNA splicing in islets from STF-treated Akita mice. As shown in **Figure 3A**, the level of *XBP1-s* mRNA was significantly reduced in islets from STF group relative to vehicle group, as assessed by RT-PCR followed by electrophoretic separation. This result was corroborated by qRT-PCR using Xbp1 splicing-specific primers (**Figure 3B**), whereas *XBP1-t* mRNA levels were only moderately affected (**Figure 3C**). We further detected that the mRNA levels of XBP1 target genes *EDEM1*, *P58*, and *Bip* were highly suppressed in islets from STF-treated mice (**Figures 3D–F**). Moreover, the transcript levels of generic RIDD targets of IRE1-α—Col61a and Blos1— were suppressed in the Akita islets; however, their levels were significantly reversed in islets from STF-treated mice (**Figures 3G, H**). In addition, under ER stress, insulin 1 and insulin 2 mRNAs are known to be cleaved directly by IRE1-α RNase activity as β-cell-specific RIDD targets (10, 56). Both insulin 1 and insulin 2 mRNAs were expectedly down-regulated in Akita islets (**Figures 3I, J**). However, STF treatment significantly reversed their expression in the Akita islets (**Figures 3I, J**). Together, our results reveal that STF treatment suppresses Akita mutation-induced IRE-1α activation in Akita islets. Notably, STF at the dose of 10 mg/kg BW reversed hyperactivated IRE1α activity to normal level but did not completely abolish IRE-1α activity (**Figures 3A, B**). Interestingly, although STF is known to inhibit IRE-1α activity only, our results revealed that STF also suppressed the Akita mutation-induced increase in *ATF4* and *CHOP* mRNA levels, key components of the PERK pathway (**Figures S2A, B**). The effect of STF on PERK pathway could be due to cross-talks among the branches of UPR under *in vivo* conditions as previously reported (57, 58).

### STF Promotes β-cell Viability in Akita Mice

**Figure 4:**
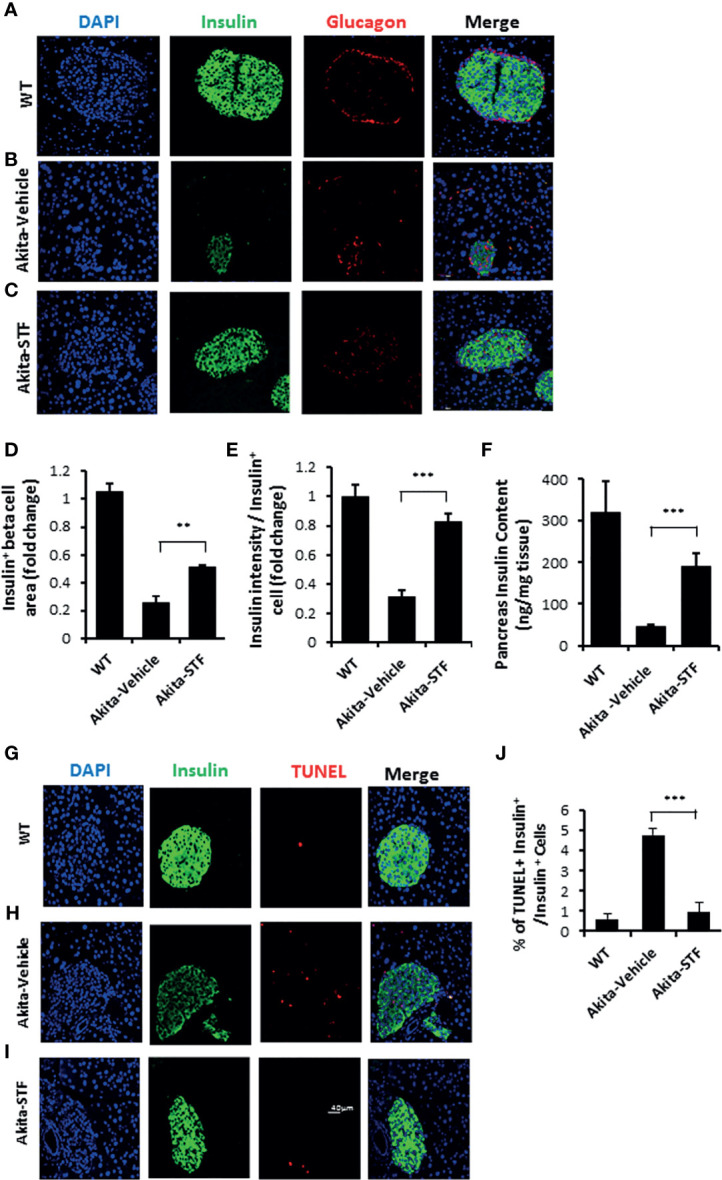
STF preserves β-cell mass and viability and suppresses β-cell apoptosis in Akita mice. **(A-C)** Immunofluorescence staining of pancreatic sections. Pancreases were sectioned and slides were stained with anti-insulin antibody (green, β-cell marker), anti-glucagon antibody (red, α-cell marker), and DAPI (blue). Slides were imaged with an Olympus FV1000 confocal microscope. **(D)** Quantification of insulin^+^ β-cell area after normalized to that for C57B/6 mice. **(E)** Insulin staining intensity. The average insulin staining intensity was quantified using ImageJ and normalized with that for C57B/6 mice designated as 1. **(F)** Insulin content measurement by ELISA as detailed in Methods and Materials. **(G–I)** TUNEL staining in pancreatic sections. Pancreatic sections were stained with anti-insulin antibody (green, β-cell marker), TUNEL (red, cell death), and DAPI (blue). Slides were imaged with an Olympus FV1000 confocal microscope. **(J)** Quantification of percentage of TUNEL^+^ insulin^+^ β-cells/insulin^+^ cells. At least 50 islets were counted for each group. Data are the mean± SEM. **P < 0.01 and ***P < 0.001.

As diabetes progression in Akita mice is associated with gradual β-cell loss and IRE-1α is activated before the onset of diabetes in Akita mice, we investigated whether the STF improvement of diabetic conditions is associated with the protection of islet β-cells in the Akita mice. In pancreatic sections, Akita islets not only exhibited reduced β-cell mass but also significantly decreased insulin staining intensity in existing β-cells (**Figures 4A, B, D, E**), indicative of β-cell loss and dysfunction. In contrast, the STF-treated Akita mice possessed approximately twice the β-cell mass and significantly higher insulin staining intensity compared to that in vehicle-treated mice (**Figures 4B–E**). On the other hand, the α cell numbers, marked by glucagon immunostaining, remained comparable between STF and vehicle groups (**Figures 4A–C**). Consistent with these results, total pancreatic insulin content as quantified by ELISA was markedly higher in STF-treated Akita mice than their vehicle-treated counterparts (**Figure 4F**). To determine whether the increase in β-cell mass by STF could be attributed to an inhibition of islet cell apoptosis, we assessed apoptosis using TUNEL staining, a marker for apoptosis. An increase in TUNEL ^+^ insulin^+^ cells was observed in the vehicle-treated Akita mice relative to WT mice (**Figures 4G–J**). However, TUNEL staining was considerably reduced in STF-treated Akita to a level comparable to that of WT (**Figures 4G–J**). Treatment with STF also significantly reduced the number of CASP^+^ (a critical protein in the execution of apoptosis) in insulin^+^ cells of Akita islets compared to that in vehicle-treated islets (**Figures S3A–D**). In contrast, STF treatment appeared not to affect β-cell proliferation as the frequency of Ki67^+^ insulin^+^ cells remained comparable between the STF- and vehicle-treated mice (**Figure S4**). Lastly, the expression of apoptotic effector genes BAX and Bak1, pro-apoptotic inducer gene p53, and negative cell-cycle regulator p21 were significantly suppressed in the islets of STF-treated Akita mice (**Figures S5A–D**). In sum, these results indicate that inhibition of increased IRE1-α activity by STF suppresses β-cell apoptosis in Akita mice which in turn leads to a preservation of β cell mass.

### STF Improves β-Cell Function in Akita Mice

**Figure 5:**
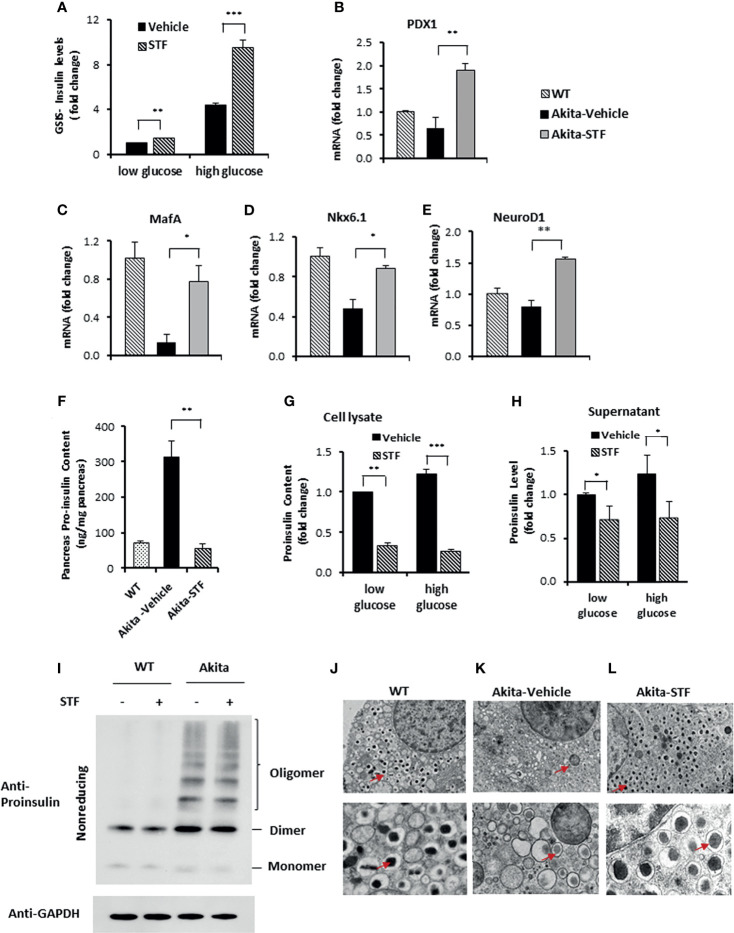
STF improves Akita islet β-cell function. **(A)** Glucose-stimulated insulin secretion of 20 islets isolated from Akita mice treated with STF or vehicle and incubated with 2.5 mM and 16.7 mM glucose. Secreted insulin was measured by ELISA. The data was presented as fold change and normalized with total protein concentration, with the amount of insulin secreted in response to 2.5 mM glucose from vehicle-treated group set to 1.0. **(B–E)** mRNA levels for indicated genes were analyzed in islets isolated from Akita mice treated with STF or vehicle by qRT-PCR. The results are expressed as fold change and are representative of 3 independent experiments. **(F)** Proinsulin content measurement by ELISA as detailed in Methods and Materials. **(G, H)** Proinsulin content and secretion measurement. 20 islets isolated from Akita mice treated with STF or vehicle were incubated with 2.5 mM and 16.7 mM glucose. Secreted proinsulin was measured by ELISA. Proinsulin content measurement by ELISA as detailed in Methods and Materials. The data was presented as fold change and normalized with total protein concentration, with the amount of proinsulin in response to 2.5 mM glucose from vehicle-treated group set to 1.0. **(I)** Proinsulin misfolding detection by Western blotting under nonreducing condition. Islets were treated with compounds at indicated concentrations for 16 hours. The data is representative of 3 independent experiments. **(J–L)** Ultrastructure of β-cells in islets isolated from Akita treated with STF as in by transmission electron microscopy. Images at the top panel and the bottom panel were taken at 3,000x and 10,000x, respectively. Arrows point to dark mature insulin granules. *P < 0.05, **P < 0.01, and ***P < 0.001.

We interrogated whether STF also improves β-cell function. Indeed, our observations that STF heightened insulin production in β-cells (**Figures 4C–F**) and increased serum insulin levels (**Figure 2G**) suggest an improvement in Akita β-cell function. To further interrogate this, we assessed the glucose-stimulated insulin secretion in islets and found that insulin secretion was significantly higher under both basal (2.5 mM glucose concentration) and stimulated (16.7 mM glucose concentration) conditions in islets isolated from STF-treated Akita mice compared to vehicle-treated group (**Figure 5A**). Next, as high glucose and the ensuing ER stress in β-cells down-regulate the expression of β-cell-specific transcription factors (23, 59–62), which are essential for the maintenance of normal β-cell function (63–65), and there is evidence indicating β-cell dedifferentiation in Akita mice (66), we investigated the effect of STF treatment on their expression levels in Akita β-cells. As expected, the mRNA levels of several β-cell-specific transcription factors (*Pdx1*, *MafA*, *NeurD1*, and *Nkx6.1*) were significantly down-regulated in Akita islets compared to WT islets (**Figures 5B–E**). Notably, their reduced expression was markedly reversed in islets of Akita mice treated with STF (**Figures 5B–E**).

Akita mutant proinsulin tends not only to misfold but also forms heterogeneous complex with WT proinsulin, thus entrapping WT proinsulin in the ER (34, 35, 67) and limiting bioactive insulin production and secretion, thus leading to ER stress and β-cell dysfunction and death (44, 68). The disturbed ER environment in turn further exacerbates proinsulin misfolding (69). We therefore investigated whether the increased insulin production (**Figures 2G**, **4C–F**, **5A**) seen with STF treatment is associated with reduced proinsulin levels. As expected, proinsulin content was dramatically and significantly increased in the Akita mouse pancreas relative to WT pancreas, but was reversed to normal level in the STF-treated Akita pancreas (**Figure 5F**). Of note, this effect of STF on proinsulin is opposite to that seen for insulin (**Figure 4F**). While the STF suppression of proinsulin level in Akita islets can be interpreted as an outcome of STF-improved ER environment which permits more proinsulin conversion to mature insulin; it is also possible that this effect is mediated through increased proinsulin release from β cells or through modulation of proinsulin misfolding. To address whether STF increases proinsulin secretion, we analyzed proinsulin levels in Akita islets from Akita mice treated with STF or vehicle. We found that proinsulin content (cell lysate) and secretion (supernatant) were both attenuated in STF group, under either basal (2.5 mM) or high (16.7 mM) glucose concentration (**Figures 5G, H**). To address whether STF affects proinsulin misfolding, we examined proinsulin folding status in Akita islets under nonreducing condition. Consistent with previous reports (42), a significant port of the proinsulin in nonreduced lysates of Akita islets was detected as high molecular weight oligomers relative to WT islets (**Figure 5I**). STF treatment of Akita islets showed no apparent effect on proinsulin oligomers (**Figure 5I**).

Finally, as mature insulin is formed from proinsulin processed in the Golgi complex and stored in secretory granules for release, we examined the effect of STF on insulin secretory granules and ultrastructure of islet β-cells using transmission electron microscopy. Our results showed that whereas there was marked reduction in the number of dark electron dense-core granules (mature insulin granules) and increase in the number of light or “gray” electron dense-core granules (immature insulin granules) in Akita β-cells compared to WT β-cells (**Figures 5J–L**), β-cells in the STF-treated Akita mice exhibited a marked increase in dense-core insulin granules and (**Figure 5L**), similar to those seen in the WT islets (**Figure 5J**). Together, these results demonstrate that STF normalization of IRE-1α activity facilitates insulin granule formation.

### STF Suppresses ER Stress-Related Inflammation and Oxidative Stress in Akita Islets

**Figure 6:**
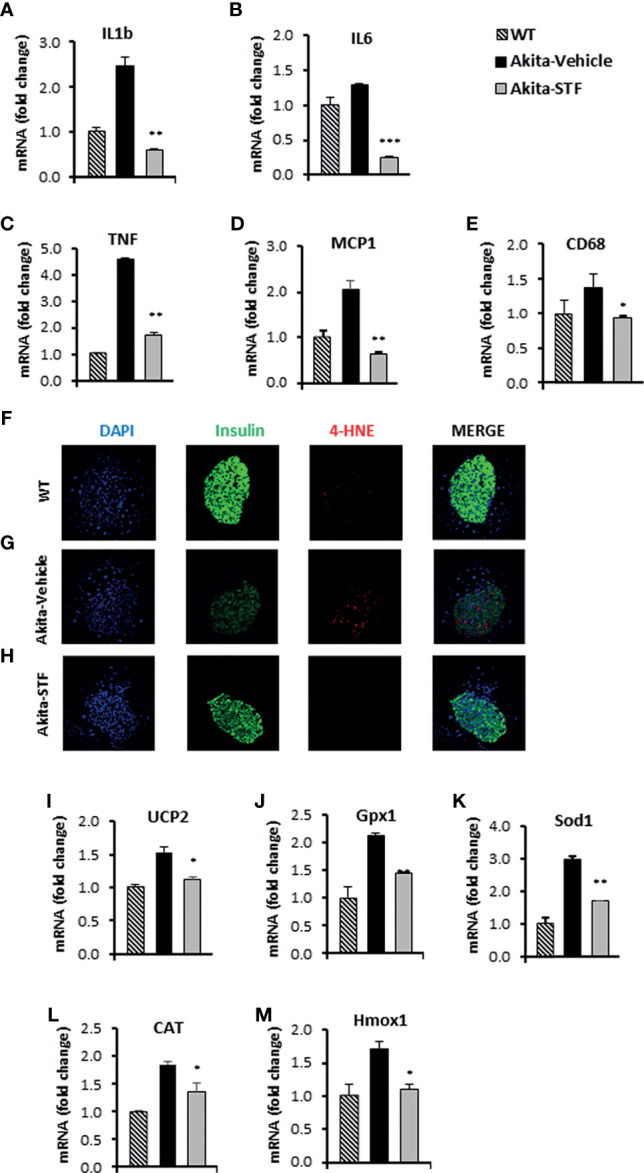
STF attenuates the ER stress-associated inflammation and oxidative stress. **(A–E)** mRNA levels for indicated genes involving inflammation were analyzed in islets isolated from Akita mice treated with STF or vehicle (as in) by qRT-PCR. The results are expressed as the fold-increase over mRNA levels and are representative of 3 independent experiments. **(F–H)** Immunofluorescent staining in pancreatic sections. Pancreatic sections were stained with anti-insulin antibody (green, β-cell marker), 4-HNE (red, oxidative stress), and DAPI (blue). Slides were imaged with an Olympus FV1000 confocal microscope. **(I–M)** mRNA levels for indicated anti-oxidant genes were analyzed in islets isolated from Akita mice treated with STF or vehicle by qRT-PCR. The results are expressed as fold change and are representative of 3 independent experiments. *P < 0.05, **P < 0.01, and ***P < 0.001 compared to Akita-vehicle group. Bars indicate SEM.

ER stress has been shown to cause and potentiate inflammation and oxidative stress that cooperatively contribute to ER stress-mediated cell death (70). We therefore investigated whether STF treatment affects these processes in the Akita islets. We found that mRNA levels of the ER stress-associated pro-inflammatory cytokine genes IL-1β, IL6, and TNF were increased in the Akita islets compared to WT islets (**Figures 6A–C**). We also detected increased transcript levels of MCP1 and CD68 (**Figures 6D, E**), markers that are highly expressed in tissue monocytes and macrophages, respectively. Strikingly, STF corrected the mRNA levels of these genes to normal levels in the Akita islets (**Figures 6A–E**).

Next, we assessed the effect of STF on oxidative stress in Akita islets. We observed an obvious nuclear accumulation of the lipid peroxidation product 4-hydroxynonenal (4-HNE), a marker of oxidative stress, in the Akita islets compared to WT islets (**Figures 6F, G**). In addition, the mRNA levels of several antioxidant genes, including those encoding the mitochondrial uncoupling protein 2 (UCP2), glutathione peroxidase 1 (Gpx1), superoxide dismutase 1 (Sod1), catalase (CAT), and heme oxygenase 1 (Hmox1), were up-regulated in the Akita islets relative to WT islets (**Figures 6I–M**), reflecting a compensatory mechanism of anti-oxidation through the antioxidant gene up-regulation (71, 72). Notably, the nuclear accumulation of 4-HNE and up-regulation of antioxidant genes were abolished in the islets of Akita mice treated with STF (**Figures 6G, H**).

### Ire1-α RNase Inhibitor 4μ8C Ameliorates Diabetic Conditions in Akita Mice

**Figure 7:**
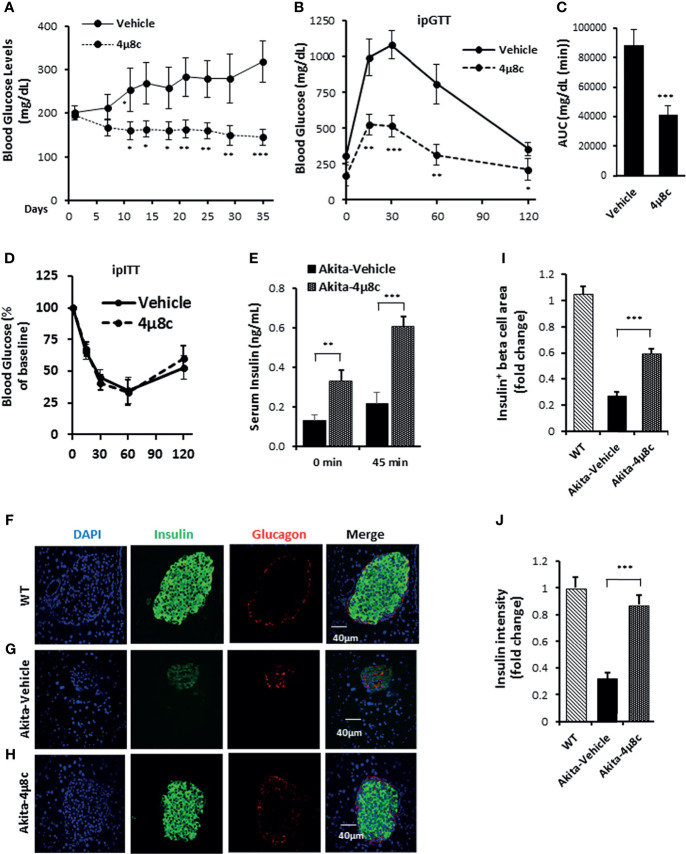
4μ8C ameliorates diabetic conditions of Akita mice and inhibits IRE1α RNase activity. **(A)** Fasting blood glucose levels were measured in Akita mice treated with vehicle (n = 7) or 4μ8C (n = 6) at indicated time points. **(B, C)** Glucose tolerance test. Blood glucose levels **(B)** measured at indicated time points after intraperitoneal injection of glucose (1.5g/kg body weight) following 6-h fasting and the AUC (area under the curve, **C**). **(D)** Insulin tolerance test. Blood glucose levels measured at indicated time points after intraperitoneal injection of insulin (0.75 IU/kg body weight) following 4-h fasting. **(E)** In vivo glucose-stimulated insulin secretion. Serum insulin levels measured at indicated time points after intraperitoneal injection of glucose (1.5g/kg body weight) following 6-h fasting. **(F–H)** Immunofluorescence staining of pancreatic sections. Pancreases were sectioned and slides were stained with anti-insulin antibody (green, β-cell marker), anti-glucagon antibody (red, α-cell marker), and DAPI (blue). Slides were imaged with an Olympus FV1000 confocal microscope. **(I)** Quantification of insulin^+^ β-cell area. Total area of all islets per section was calculated from a total of six sections for each of three mice using insulin^+^ cells to demarcate islet β-cells and normalized with that for C57B/6 mice designated as 1. **(J)** Insulin staining intensity. The average insulin staining intensity was quantified using ImageJ and normalized with that for C57B/6 mice designated as 1. *P < 0.05, **P < 0.01, and ***P < 0.001. Bars indicate SEM.

To ascertain that STF improves diabetic conditions of Akita mice *via* inhibition of IRE1α, we utilized another structurally distinct IRE1α RNase inhibitor 4μ8C (73) for the efficacy studies. Treatment with 4μ8C improved fasting blood glucose levels in Akita mice while vehicle-treated Akita mice showed a progressive rise in blood glucose level (**Figure 7A**). 4μ8C treatment also significantly improved glucose tolerance in Akita mice (**Figures 7B, C**), with no apparent difference in body weight (**Figure S6**) or insulin tolerance (**Figure 7D**), compared to vehicle-treated Akita mice. Moreover, there was a marked increase in serum insulin levels and pancreatic insulin content in Akita group treated with 4μ8C (**Figures 7E**, **S7**). 4μ8C treatment also significantly preserved the β-cell area and restored insulin staining intensity in β-cells (**Figures 7F–J**). Furthermore, 4μ8C treatment significantly alleviated the Akita mutation-induced increase in *XBP1* splicing (**Figures S8A, B**). 4μ8C also attenuated the heightened mRNA levels of the XBP1-s target genes Grp94, Bip and P58 in Akita islets (**Figures S8C–E**) and significantly reversed the repressed levels of RIDD target mRNAs Blos1, Col6a1, and INS1 in Akita islets (**Figures S8F–H**). Lastly, 4μ8C attenuated the increased levels of the PERK pathway genes ATF4 and CHOP in Akita islets (**Figures S8I, J**). Together, we conclude that both STF and 4μ8C are able to correct the diabetic conditions of Akita mice likely through the inhibition of Ire1α RNase activity.

## Discussion

In this study, we observed that IRE-1α activity was progressively up-regulated in the islets of Akita mice in an age-dependent fashion and that the increased IRE1α activity predates the onset of diabetes in Akita mice. Importantly, we showed that two IRE-1α RNase inhibitors STF and 4μ8c markedly ameliorated the diabetic conditions and protected β-cell viability and function in Akita mice, thus revealing IRE-1α as an important target in β-cell protection and diabetes therapy.

In further pursuit of how the inhibition of Ire1α RNase activity protects against β-cell dysfunction and loss in Akita mice, we examined the effect of STF on ER stress/UPR. We discovered that not only was Ire1α RNase activity reduced, as expected, but also the insulin misfolding-induced activation of PERK pathway was suppressed, which likely reflects the cross talk among different UPR pathways (74, 75). Additionally, the up-regulated levels of inflammation and oxidative stress in Akita islet cells were suppressed by the treatment of Ire1α inhibitor. Therefore, our data reveal the amelioration of ER stress and downstream inflammation and oxidative stress as the underlying mechanisms of the protection of β-cell dysfunction and demise in Akita mice by inhibiting Ire1α activity.

Previous studies have reported that imatinib and similar tyrosine kinase inhibitors exhibit β cell protection by inhibiting IRE1α kinase activity, either directly or through an intermediary factor, leading to the attenuation of IRE1α RNase activity (11, 13, 14). However, given the promiscuous nature of kinase inhibitors which generally target multiple kinases, it is possible that IRE1α might not be the sole kinase target or even the cellular target of imatinib and related tyrosine kinase inhibitors for their biological activities. Indeed, for example, KIRA6, a small molecule published as an IRE1α kinase inhibitor (11), was found to potently inhibit the activity of over 60 kinases by >70% attenuation among 220 kinases tested (15, 16). In addition to target kinases, KIRA6 was also discovered to bind a large number of nonkinase nucleotide-binding proteins by photoaffinity labeling approach (17). Similarly, imatinib has also been reported to serve as a partial agonist of peroxisome proliferator-activated receptor gamma (PPAR*γ*) (18, 19) and a modulator of autophagy (20–22), both of which protect β cell function and viability (76–79). Therefore, the effect of KIRA6, imatinib, or related tyrosine kinase inhibitors on β cell protection is mostly likely the outcome of acting on multiple factors in addition to (if any) IRE1α inhibition. Moreover, IRE1α also directly activates signaling pathways such as JNK/ASK1-MAPK pathways, which regulates cell death and fate (80, 81), independently of its RNase domain. Therefore, even if the above kinase inhibitors do engage IRE1α for their effects, it is still unknown whether the inhibition of IRE1α RNase function is critical in ameliorating the diabetic condition. Our current work provides clear evidence that inhibiting IRE1α RNase activity alone with two different IRE1α RNase inhibitors STF and 4μ8c is sufficient to significantly improve the diabetic condition and β cell function and health in Akita mice. While we cannot rule out the possibility that these compounds might have other targets that are responsible for or contributing to the improvement of diabetes, we consider such a possibility unlikely as these two different compounds would have most likely engaged in different unknown targets if not the known target IRE1α. In addition, these compounds have been shown to provide benefits in other disease models as IRE1α RNase inhibitors (47, 48).

IRE1α has been documented to serve an important modulatory role in multiple physiological contexts including β cell function, growth and survival, which poses a question as to whether IRE1α inhibition would actually improve β cell function and survival and diabetic conditions under ER stress-related situations. Our results provided insights to this question by showing that pharmacological inhibition of IRE1α markedly ameliorates diabetic condition and improves β cell mass and function in the Akita diabetes mice. Of note, unlike a genetic knockout, pharmacological inhibition of IRE1α of appropriate dose does not totally abolish the IRE1α function but instead reverses the hyperactivated IRE1α back to basal level as shown by our data. In turn, the IREα hyperactivation-induced diabetic conditions in Akita mice are corrected without the unwanted side effects that are associated with IREα knockout. Our findings therefore highlight the notion that the normalization (not elimination) of IRE1α activity as the key to an effective therapeutic use of pharmacological inhibitors on proteins with physiologically important but pathologically heightened activity.

## Conclusions

In summary, our studies showed that IRE1α RNase inhibitors STF and 4μ8c preserves β-cells and prevents the development of diabetes in insulin protein misfolding-causing Akita mice. This protection is associated with significant increase in the number of β-cells through the attenuation of apoptosis and the preservation of β-cell function including basal and glucose-stimulated insulin secretion. IRE1α inhibitors achieved these effects through the suppression of ER stress-induced excessive activation of UPR. These findings may offer an effective therapeutic strategy for MIDY patients. In addition, as ER stress and insulin misfolding are well established in their roles in β cell dysfunction and demise in type 2 diabetes, IRE1α inhibition may well be considered for the treatment of type 2 disease.

## Data Availability Statement

The original contributions presented in the study are included in the article/**Supplementary Material**. Further inquiries can be directed to the corresponding author.

## Ethics Statement

The animal study was reviewed and approved by Institutional Animal Care and Use Committee of the University of Oklahoma Health Science Center.

## Author Contributions

OH-P, VE, and RU generated research data. H-YL designed the research project, contributed to discussion, and reviewed/edited the manuscript. WW conceived, initiated, and designed the research project, reviewed the data, and wrote the manuscript. WW is the guarantor of this work and, as such, had full access to all the data in the study and takes responsibility for the integrity of the data and the accuracy of the data analysis. All authors contributed to the article and approved the submitted version.

## Funding

This work was supported by Oklahoma Center for the Advancement of Science and Technology and National Institutes of Health (Grants GM103636, DK108887, DK116017) to WW.

## Conflict of Interest

The authors declare that the research was conducted in the absence of any commercial or financial relationships that could be construed as a potential conflict of interest.

## Publisher’s Note

All claims expressed in this article are solely those of the authors and do not necessarily represent those of their affiliated organizations, or those of the publisher, the editors and the reviewers. Any product that may be evaluated in this article, or claim that may be made by its manufacturer, is not guaranteed or endorsed by the publisher.
