# Supplementary material for: Pharmacological Inhibition of Inositol-Requiring Enzyme 1α RNase Activity Protects Pancreatic Beta Cell and Improves Diabetic Condition in Insulin Mutation-Induced Diabetes
Source: Front Endocrinol (Lausanne). 2021 Oct 5;12:749879. doi: 10.3389/fendo.2021.749879 (PMC8524045; doi:10.3389/fendo.2021.749879)
Supplement: Supplementary file 1 [file DataSheet_1.docx]

**Supplemental Figure 1.**

**A-C**. mRNA levels for indicated genes were analyzed in islets isolated from Akita mice or age-matched C57B/6 mice by qRT-PCR. The results are expressed as the fold change over mRNA levels in respective age-matched controls (represented by the dashed line) and are representative of 3 independent experiments. * P < 0.05, ** P <0.01, and *** P <0.001. Bars indicate SEM.

**Supplemental Figure 2.**

**A-B**. mRNA levels for indicated anti-oxidant genes were analyzed in islets isolated from Akita mice treated with STF or vehicle by qRT-PCR. The results are expressed as fold change and are representative of 3 independent experiments. * P < 0.05, ** P <0.01, and *** P <0.001 compared to Akita-vehicle group. Bars indicate SEM.

**Supplemental Figure 3.**

**A-C**. Immunofluorescence staining of pancreatic sections. Pancreases sections were stained with an-Casp3 antibody (red), anti-insulin antibody (green, β-cell marker), and DAPI (blue). Slides were imaged with an Olympus FV1000 confocal microscope. **D**. Quantification of percentage of Casp3^+^ insulin^+^ β-cells/insulin^+^ cells. At least 50 islets were counted for each group. Data are the mean±SEM. *** P <0.001.

**Supplemental Figure 4.**

**A**. Immunofluorescence staining of pancreatic sections. Pancreases sections were stained with an-Ki67 antibody (red), anti-insulin antibody (green, β-cell marker), and DAPI (blue). Slides were imaged with an Olympus FV1000 confocal microscope. **B**. Quantification of percentage of Ki67^+^ insulin^+^ β-cells/insulin^+^ cells. At least 50 islets were counted for each group. Data are the mean±SEM. NS, P >0.05.

**Supplemental Figure 5.**

**A-D**. mRNA levels for indicated anti-oxidant genes were analyzed in islets isolated from Akita mice treated with STF or vehicle by qRT-PCR. The results are expressed as fold change and are representative of 3 independent experiments. * P < 0.05, ** P <0.01, and *** P <0.001 compared to Akita-vehicle group. Bars indicate SEM.

**Supplemental Figure 6.**

Body weight of Akita mice treated with vehicle or 4μ8C.

**Supplemental Figure 7.**

Insulin content measurement of pancreatic tissues treated with vehicle or 4μ8C by ELISA as detailed in Methods and Materials.

**Supplemental Figure 8.**

**A-J**. mRNA levels for indicated anti-oxidant genes were analyzed in islets isolated from Akita mice treated with STF or vehicle by qRT-PCR. The results are expressed as fold change and are representative of 3 independent experiments. * P < 0.05, ** P <0.01, and *** P <0.001 compared to Akita-vehicle group. Bars indicate SEM.
