# Supplementary material for: Pharmacological Inhibition of Inositol-Requiring Enzyme 1α RNase Activity Protects Pancreatic Beta Cell and Improves Diabetic Condition in Insulin Mutation-Induced Diabetes
Source: Front Endocrinol (Lausanne). 2021 Oct 5;12:749879. doi: 10.3389/fendo.2021.749879 (PMC8524045; doi:10.3389/fendo.2021.749879)
Supplement: Supplementary file 2 [file Presentation_1.pptx]

## Slide 1
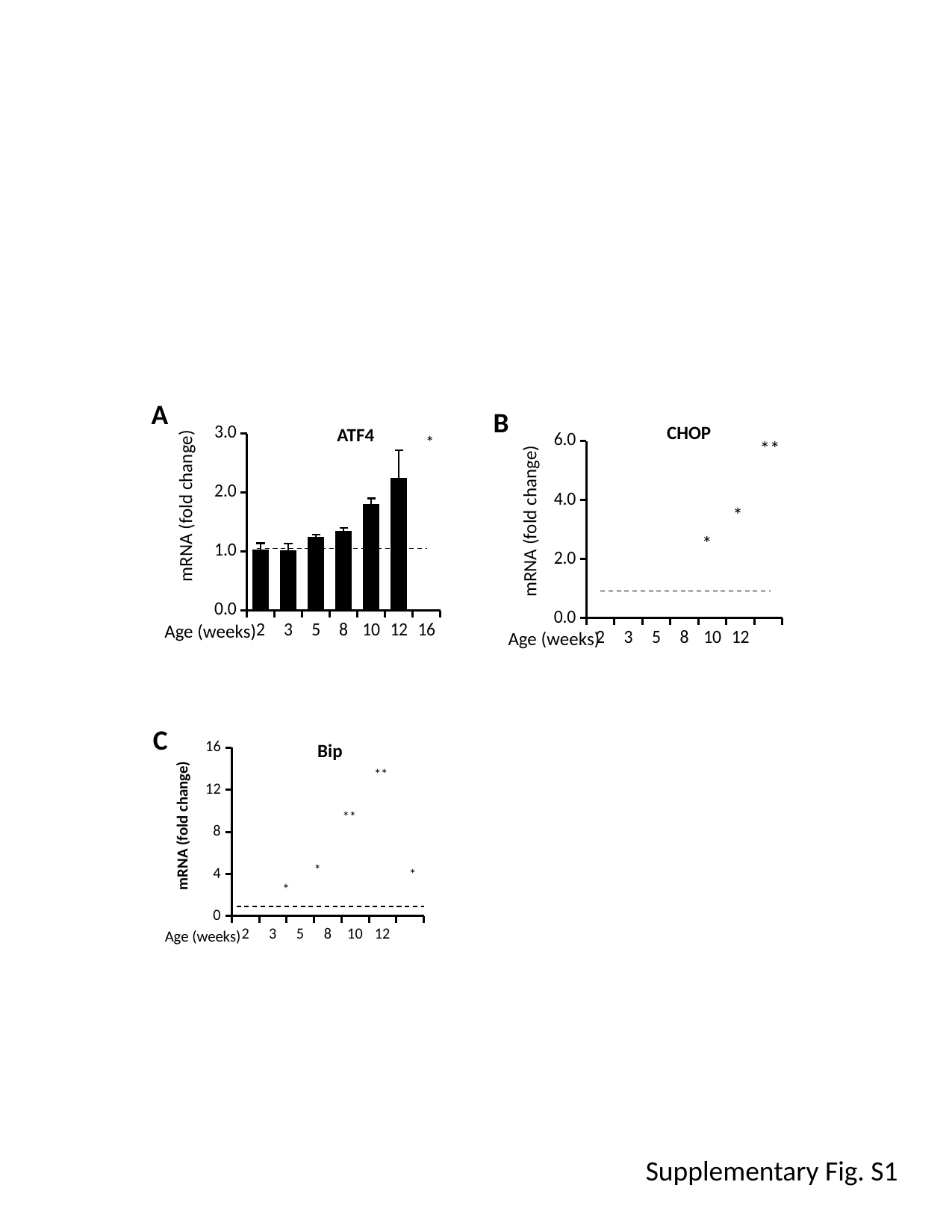

### Chart
| Category |
|---|mRNA (fold change)
Age (weeks)
[unsupported chart]
*
*
*
ATF4
A
### Chart
| Category |
|---|mRNA (fold change)
Age (weeks)
B
### Chart
| Category | Akita-Het |
|---|---|
| 2.0 | 1.45025551671282 |
| 3.0 | 1.126031592868153 |
| 5.0 | 1.374077107277871 |
| 8.0 | 2.023282184469964 |
| 10.0 | 2.95365750609951 |
| 12.0 | 5.023934326668273 |**
*
*
CHOP
### Chart
| Category |
|---|mRNA (fold change)
Age (weeks)
C
### Chart
| Category | Akita-Het |
|---|---|
| 2.0 | 1.130924443536148 |
| 3.0 | 1.592515161057372 |
| 5.0 | 3.471569103785117 |
| 8.0 | 7.865769388737902 |
| 10.0 | 12.34669010013391 |
| 12.0 | 3.061395596908244 |**
**
*
*
*
Bip
Supplementary Fig. S1

## Slide 2
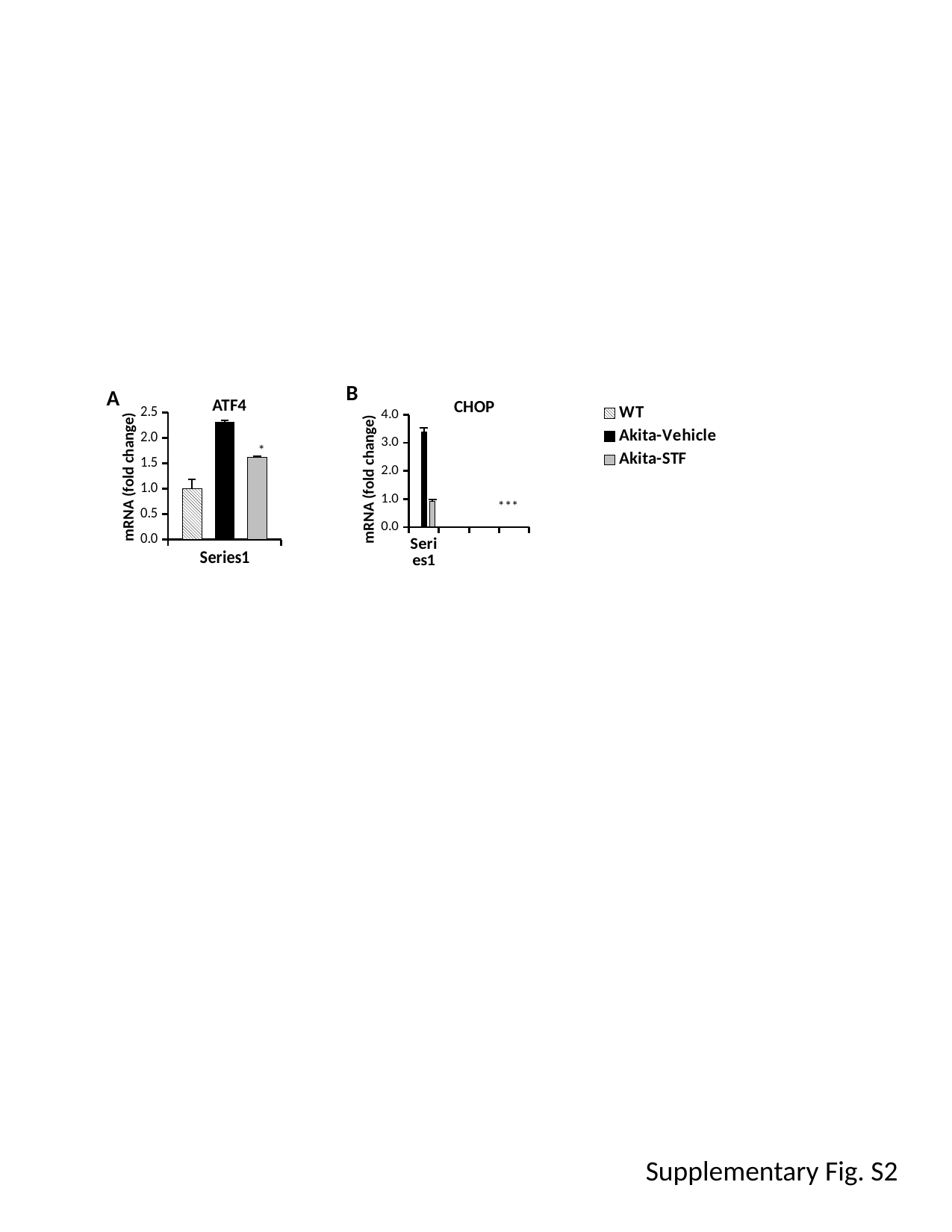

B
CHOP
mRNA (fold change)
### Chart
| Category | WT | Akita vehicle | Akita STF |
|---|---|---|---|
| | 1.000006005668685 | 3.389910785293772 | 0.922183379128633 |***
A
ATF4
mRNA (fold change)
### Chart
| Category | WT | Akita vehicle | Akita STF |
|---|---|---|---|
| | 1.001736138643228 | 2.299066396351987 | 1.614233392561587 |*
### Chart
| Category | WT | Akita-Vehicle | Akita-STF |
|---|---|---|---|
| | 1.000486498118212 | 0.316982545486555 | 1.057786252944868 |Supplementary Fig. S2

## Slide 3
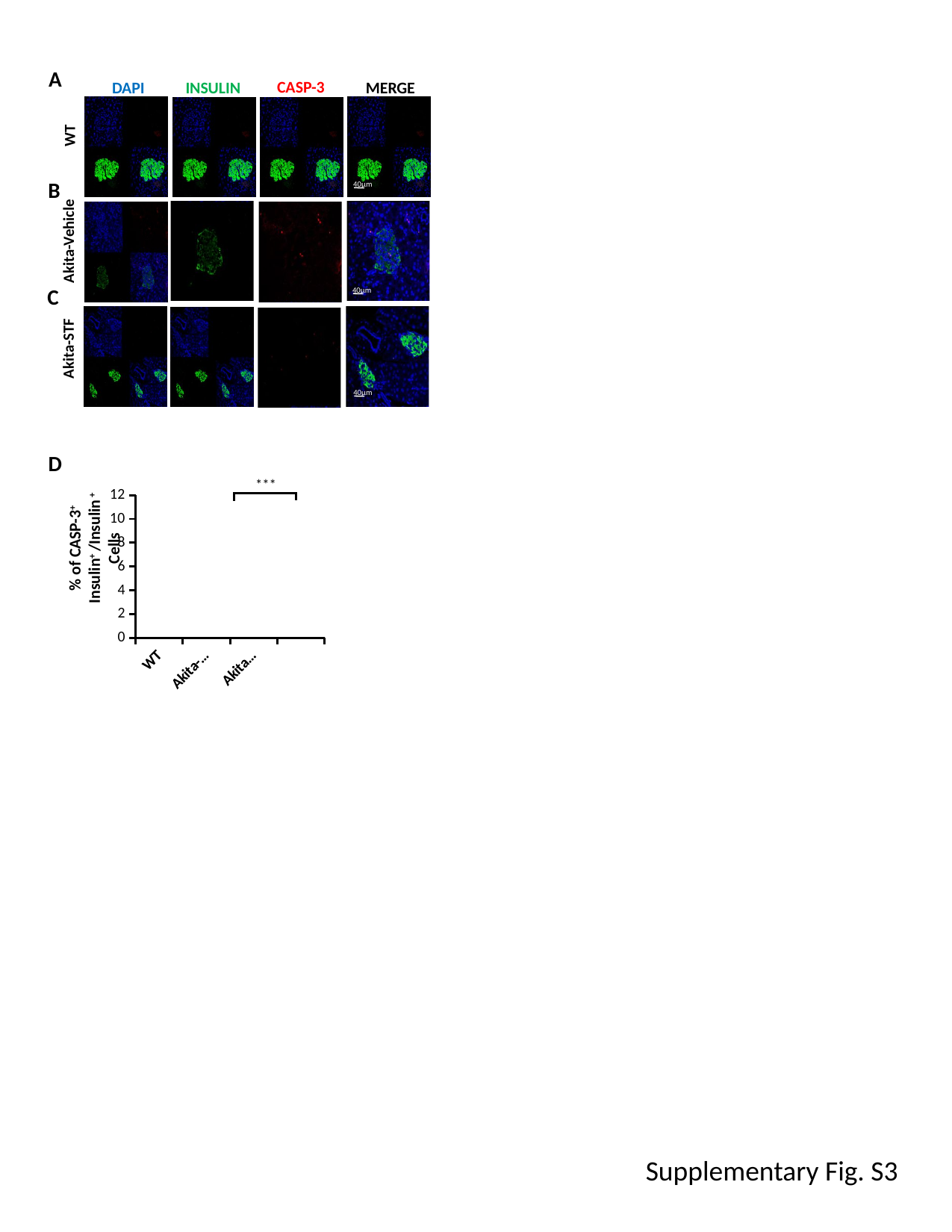

A
CASP-3
MERGE
INSULIN
DAPI
40µm
40µm
40µm
WT
B
Akita-Vehicle
C
Akita-STF
D
***
### Chart
| Category | caspase 3positive |
|---|---|
| WT | 0.834326579261025 |
| Akita-Vehicle | 6.120651695288418 |
| Akita-STF | 1.319381255686988 |% of CASP-3+ Insulin+ /Insulin + Cells
Supplementary Fig. S3

## Slide 4
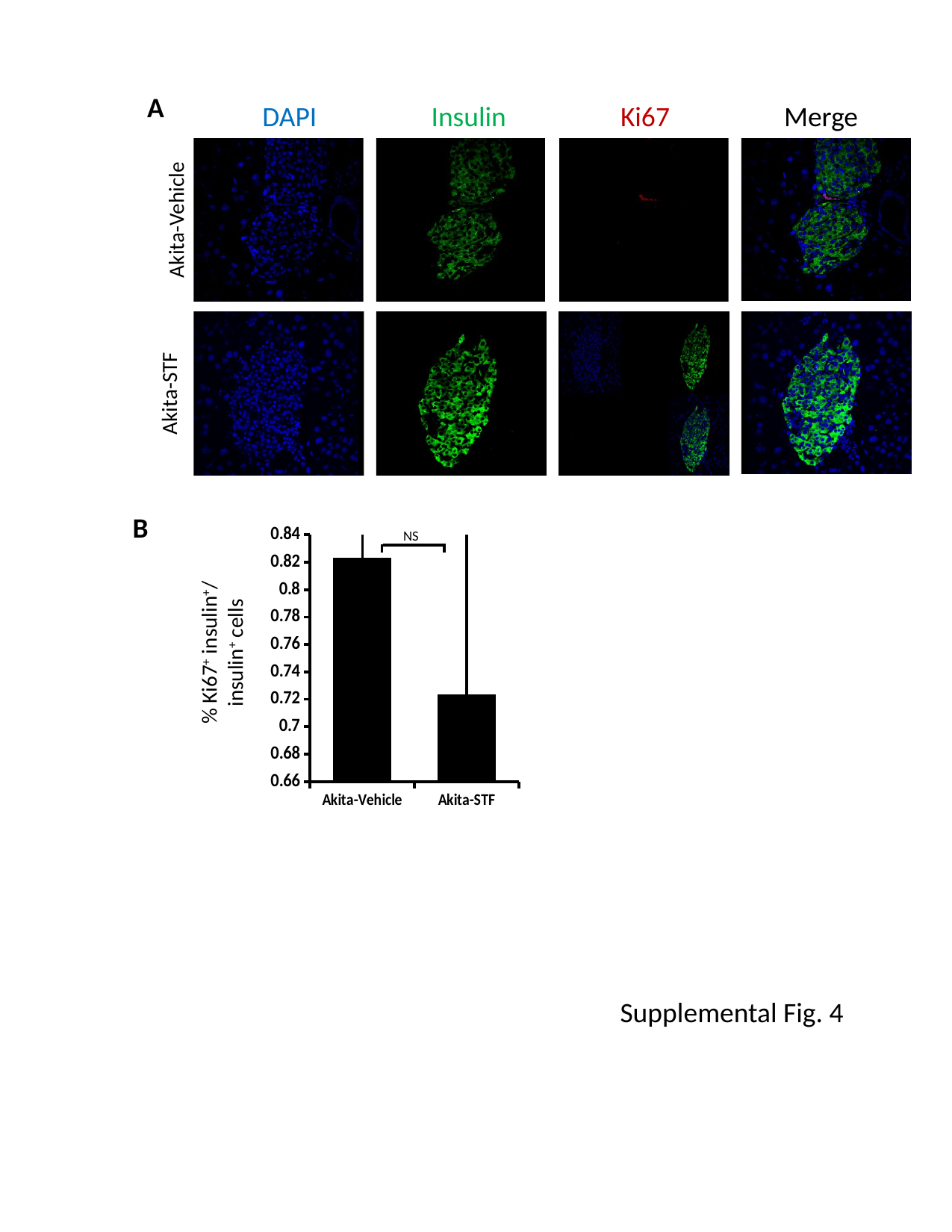

A
DAPI Insulin Ki67 Merge
Akita-Vehicle
Akita-STF
B
### Chart
| Category | insulin |
|---|---|
| Akita-Vehicle | 0.823045267489712 |
| Akita-STF | 0.723764957240606 |NS
% Ki67+ insulin+/ insulin+ cells
Supplemental Fig. 4

## Slide 5
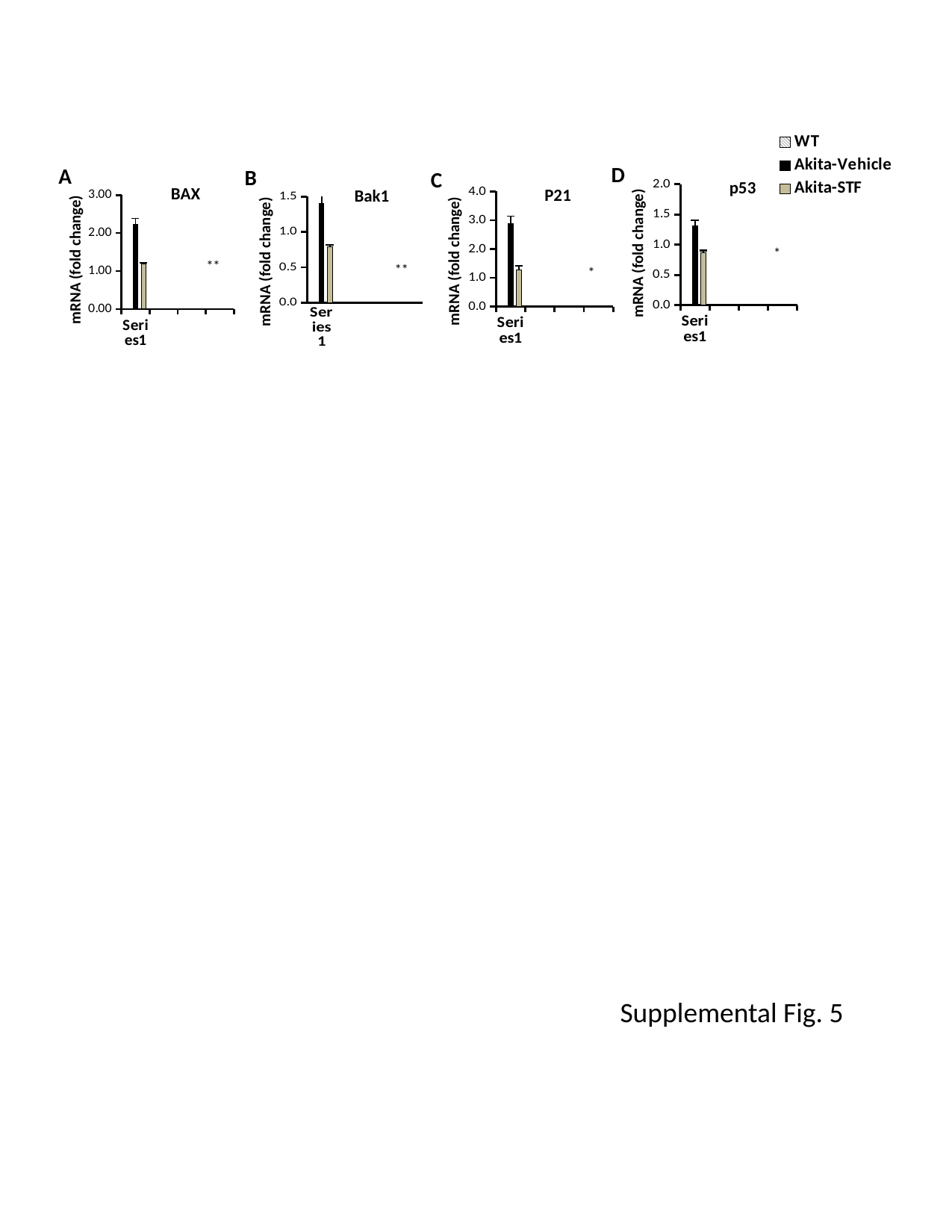

### Chart
| Category | WT | Akita-Vehicle | Akita-STF |
|---|---|---|---|
| | 1.000486498118212 | 0.316982545486555 | 1.057786252944868 |D
### Chart
| Category | WT | Akita vehicle | Akita STF |
|---|---|---|---|
| | 1.001736138643228 | 1.315582477383467 | 0.861732436026225 |mRNA (fold change)
*
p53
A
### Chart
| Category | WT | Akita vehicle | Akita STF |
|---|---|---|---|
| | 1.001946465833686 | 2.220751754846714 | 1.189664230798676 |BAX
mRNA (fold change)
**
B
Bak1
### Chart
| Category | WT | Akita vehicle | Akita STF |
|---|---|---|---|
| | 1.001946465833686 | 1.400796423510217 | 0.787691000418256 |mRNA (fold change)
**
C
### Chart
| Category | WT | Akita vehicle | Akita STF |
|---|---|---|---|
| | 1.001537843643488 | 2.890789938959449 | 1.285629827696263 |P21
mRNA (fold change)
*
Supplemental Fig. 5

## Slide 6
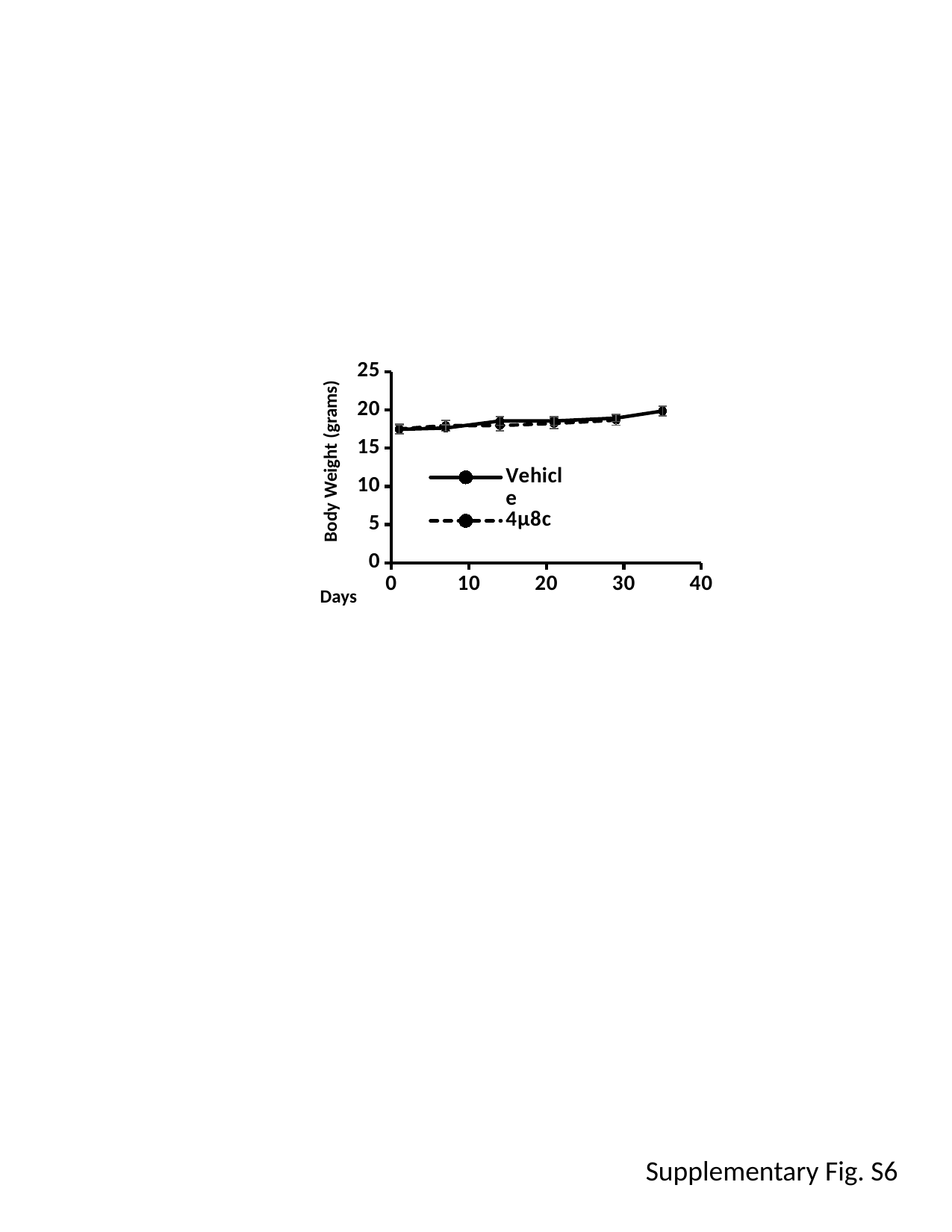

### Chart
| Category | Vehicle | 4µ8c |
|---|---|---|Body Weight (grams)
Days
Supplementary Fig. S6

## Slide 7
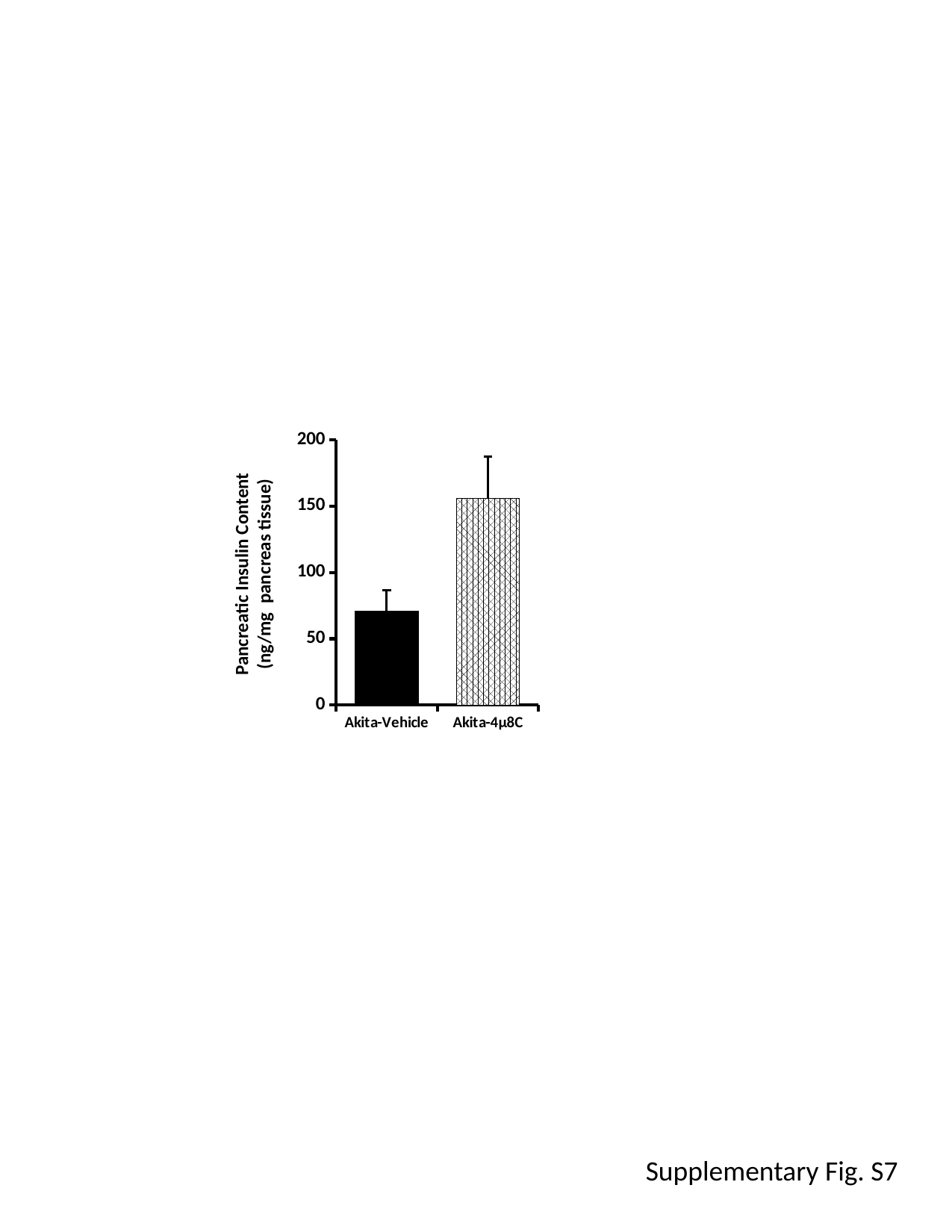

### Chart
| Category | |
|---|---|
| Akita-Vehicle | 70.85721077660185 |
| Akita-4µ8C | 156.0006144766065 |Pancreatic Insulin Content (ng/mg pancreas tissue)
Supplementary Fig. S7

## Slide 8
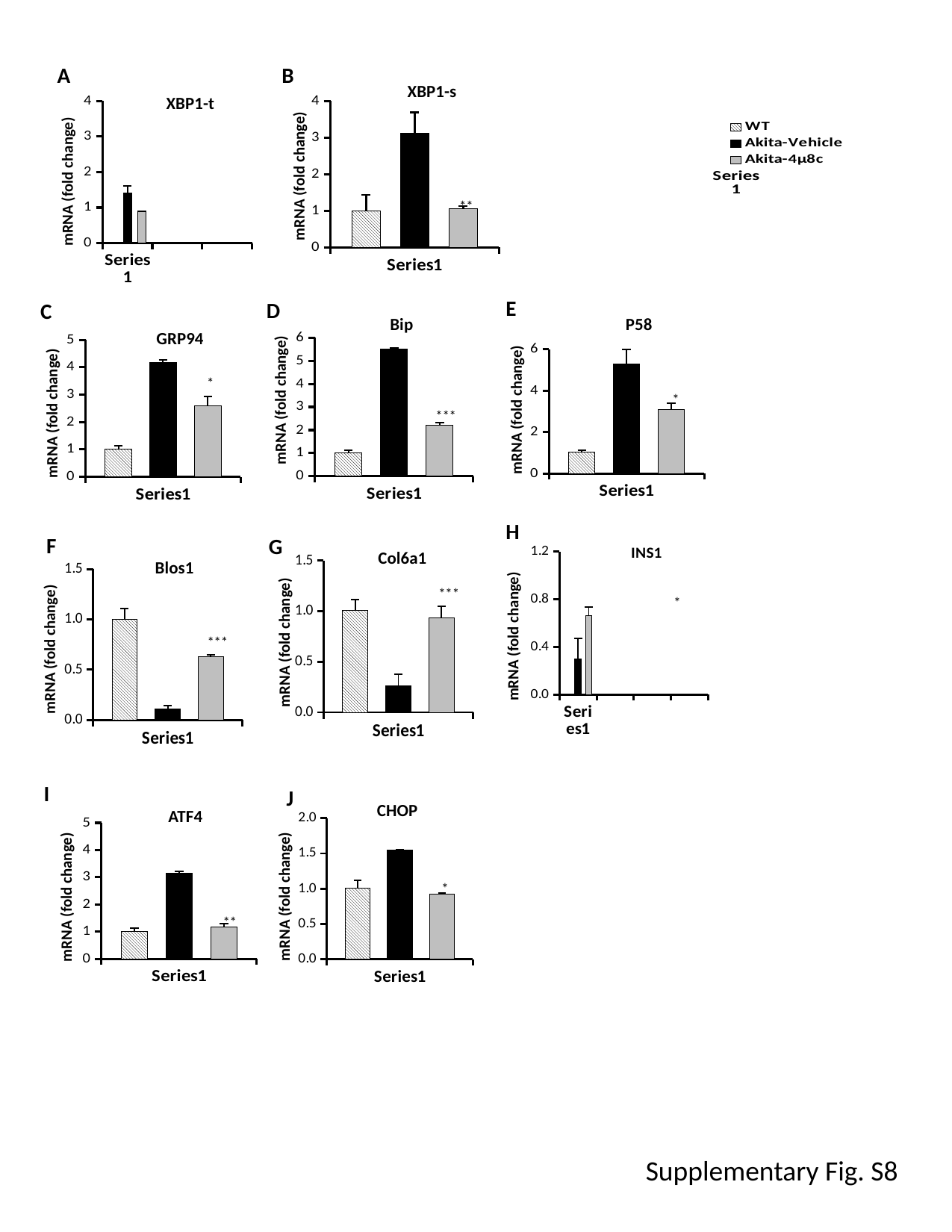

B
A
XBP1-s
mRNA (fold change)
**
### Chart
| Category | WT | Akita vehicle | Akita 4µ8C |
|---|---|---|---|
| | 1.003755887934964 | 3.11982212792795 | 1.051805955159869 |XBP1-t
### Chart
| Category | WT | Akita vehicle | Akita 4µ8C |
|---|---|---|---|
| | 1.031481535641891 | 1.41746689317786 | 0.88066975028728 |mRNA (fold change)
### Chart
| Category | WT | Akita-Vehicle | Akita-4µ8c |
|---|---|---|---|
| | 1.031481535641891 | 1.41746689317786 | 0.88066975028728 |E
D
C
P58
### Chart
| Category | WT | Akita vehicle | Akita 4µ8C |
|---|---|---|---|
| | 1.019921442728781 | 5.282572197185228 | 3.074595653149854 |*
mRNA (fold change)
Bip
### Chart
| Category | WT | Akita vehicle | Akita 4µ8C |
|---|---|---|---|
| | 1.007107690499837 | 5.507067054990683 | 2.196975250635512 |mRNA (fold change)
***
GRP94
### Chart
| Category | WT | Akita vehicle | Akita 4µ8C |
|---|---|---|---|
| | 1.020540055088998 | 4.184638244847835 | 2.590168313353622 |*
mRNA (fold change)
H
F
G
Col6a1
### Chart
| Category | WT | Akita vehicle | Akita STF |
|---|---|---|---|
| | 1.0065472987243 | 0.260220301762061 | 0.936727742121002 |***
mRNA (fold change)
### Chart
| Category | WT | Akita vehicle | Akita 4u8C |
|---|---|---|---|
| | 1.01470212154725 | 0.298374203381732 | 0.66177192313667 |*
mRNA (fold change)
Blos1
### Chart
| Category | WT | Akita vehicle | Akita STF |
|---|---|---|---|
| | 1.001177340833502 | 0.112348138927792 | 0.631067512691027 |***
mRNA (fold change)
I
J
CHOP
### Chart
| Category | WT | Akita vehicle | Akita STF |
|---|---|---|---|
| | 1.010612707606577 | 1.543544745367564 | 0.918277865363997 |*
mRNA (fold change)
ATF4
### Chart
| Category | WT | Akita vehicle | Akita 4µ8C |
|---|---|---|---|
| | 1.001791437245015 | 3.140635368772374 | 1.17006594046541 |mRNA (fold change)
**
Supplementary Fig. S8

## Slide 9
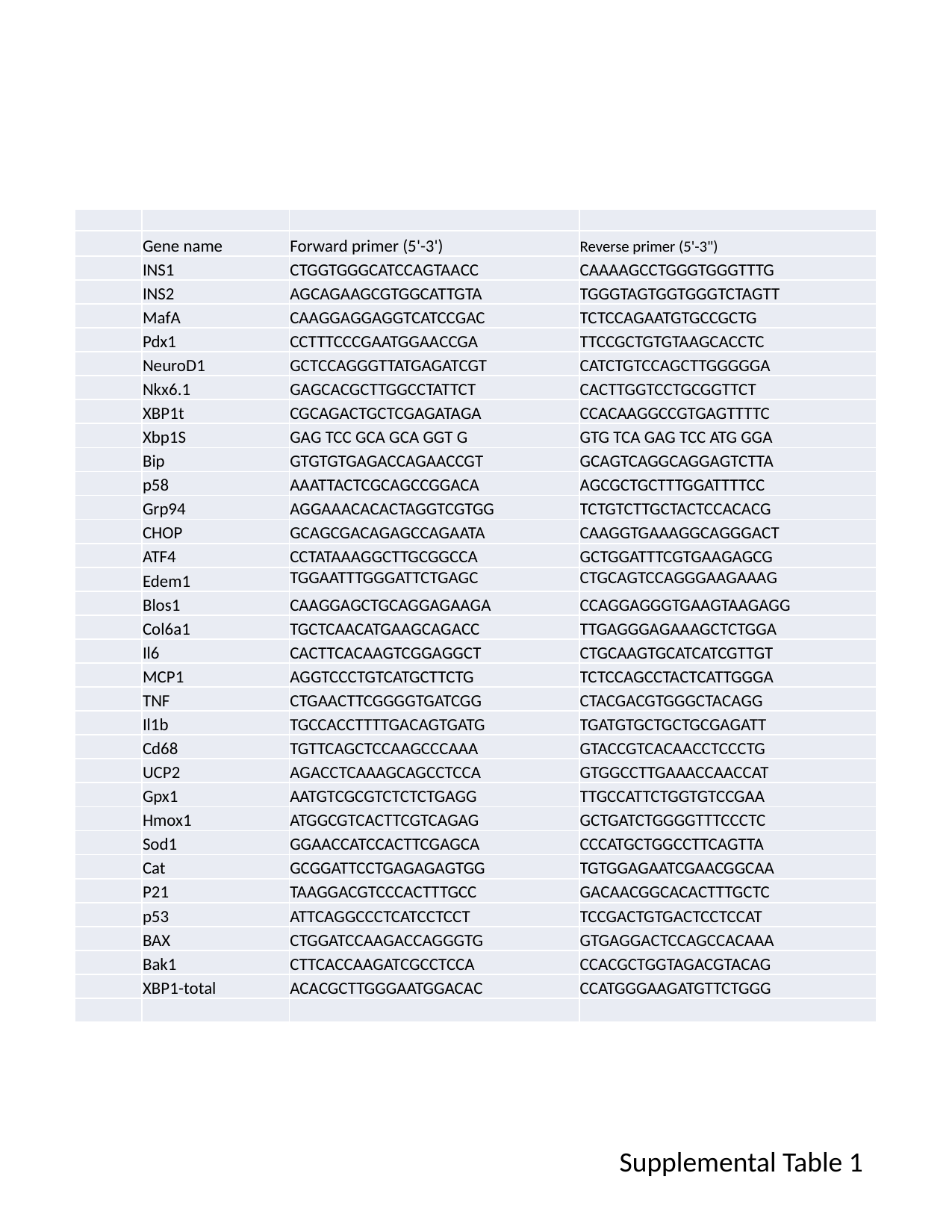

| | | | |
| --- | --- | --- | --- |
| | Gene name | Forward primer (5'-3') | Reverse primer (5'-3") |
| | INS1 | CTGGTGGGCATCCAGTAACC | CAAAAGCCTGGGTGGGTTTG |
| | INS2 | AGCAGAAGCGTGGCATTGTA | TGGGTAGTGGTGGGTCTAGTT |
| | MafA | CAAGGAGGAGGTCATCCGAC | TCTCCAGAATGTGCCGCTG |
| | Pdx1 | CCTTTCCCGAATGGAACCGA | TTCCGCTGTGTAAGCACCTC |
| | NeuroD1 | GCTCCAGGGTTATGAGATCGT | CATCTGTCCAGCTTGGGGGA |
| | Nkx6.1 | GAGCACGCTTGGCCTATTCT | CACTTGGTCCTGCGGTTCT |
| | XBP1t | CGCAGACTGCTCGAGATAGA | CCACAAGGCCGTGAGTTTTC |
| | Xbp1S | GAG TCC GCA GCA GGT G | GTG TCA GAG TCC ATG GGA |
| | Bip | GTGTGTGAGACCAGAACCGT | GCAGTCAGGCAGGAGTCTTA |
| | p58 | AAATTACTCGCAGCCGGACA | AGCGCTGCTTTGGATTTTCC |
| | Grp94 | AGGAAACACACTAGGTCGTGG | TCTGTCTTGCTACTCCACACG |
| | CHOP | GCAGCGACAGAGCCAGAATA | CAAGGTGAAAGGCAGGGACT |
| | ATF4 | CCTATAAAGGCTTGCGGCCA | GCTGGATTTCGTGAAGAGCG |
| | Edem1 | TGGAATTTGGGATTCTGAGC | CTGCAGTCCAGGGAAGAAAG |
| | Blos1 | CAAGGAGCTGCAGGAGAAGA | CCAGGAGGGTGAAGTAAGAGG |
| | Col6a1 | TGCTCAACATGAAGCAGACC | TTGAGGGAGAAAGCTCTGGA |
| | Il6 | CACTTCACAAGTCGGAGGCT | CTGCAAGTGCATCATCGTTGT |
| | MCP1 | AGGTCCCTGTCATGCTTCTG | TCTCCAGCCTACTCATTGGGA |
| | TNF | CTGAACTTCGGGGTGATCGG | CTACGACGTGGGCTACAGG |
| | Il1b | TGCCACCTTTTGACAGTGATG | TGATGTGCTGCTGCGAGATT |
| | Cd68 | TGTTCAGCTCCAAGCCCAAA | GTACCGTCACAACCTCCCTG |
| | UCP2 | AGACCTCAAAGCAGCCTCCA | GTGGCCTTGAAACCAACCAT |
| | Gpx1 | AATGTCGCGTCTCTCTGAGG | TTGCCATTCTGGTGTCCGAA |
| | Hmox1 | ATGGCGTCACTTCGTCAGAG | GCTGATCTGGGGTTTCCCTC |
| | Sod1 | GGAACCATCCACTTCGAGCA | CCCATGCTGGCCTTCAGTTA |
| | Cat | GCGGATTCCTGAGAGAGTGG | TGTGGAGAATCGAACGGCAA |
| | P21 | TAAGGACGTCCCACTTTGCC | GACAACGGCACACTTTGCTC |
| | p53 | ATTCAGGCCCTCATCCTCCT | TCCGACTGTGACTCCTCCAT |
| | BAX | CTGGATCCAAGACCAGGGTG | GTGAGGACTCCAGCCACAAA |
| | Bak1 | CTTCACCAAGATCGCCTCCA | CCACGCTGGTAGACGTACAG |
| | XBP1-total | ACACGCTTGGGAATGGACAC | CCATGGGAAGATGTTCTGGG |
| | | | |
Supplemental Table 1
